# The bioenergetic signature of isogenic colon cancer cells predicts the cell death response to treatment with 3-bromopyruvate, iodoacetate or 5-fluorouracil

**DOI:** 10.1186/1479-5876-9-19

**Published:** 2011-02-08

**Authors:** María Sánchez-Aragó, José M Cuezva

**Affiliations:** 1 1Departamento de Biología Molecular, Centro de Biología Molecular Severo Ochoa, Consejo Superior de Investigaciones Científicas-Universidad Autónoma de Madrid (CSIC-UAM), Centro de Investigación Biomédica en Red de Enfermedades Raras CIBERER-ISCIII, Instituto de Investigación Hospital 12 de Octubre, Universidad Autónoma de Madrid, 28049 Madrid, Spain

## Abstract

**Background:**

Metabolic reprogramming resulting in enhanced glycolysis is a phenotypic trait of cancer cells, which is imposed by the tumor microenvironment and is linked to the down-regulation of the catalytic subunit of the mitochondrial H^+^-ATPase (β-F1-ATPase). The *bioenergetic signature *is a protein ratio (β-F1-ATPase/GAPDH), which provides an estimate of glucose metabolism in tumors and serves as a prognostic indicator for cancer patients. Targeting energetic metabolism could be a viable alternative to conventional anticancer chemotherapies. Herein, we document that the *bioenergetic signature *of isogenic colon cancer cells provides a gauge to predict the cell-death response to the metabolic inhibitors, 3-bromopyruvate (3BrP) and iodoacetate (IA), and the anti-metabolite, 5-fluorouracil (5-FU).

**Methods:**

The *bioenergetic signature *of the cells was determined by western blotting. Aerobic glycolysis was determined from lactate production rates. The cell death was analyzed by fluorescence microscopy and flow cytometry. Cellular ATP concentrations were determined using bioluminiscence. Pearson's correlation coefficient was applied to assess the relationship between the *bioenergetic signature *and the cell death response. *In vivo *tumor regression activities of the compounds were assessed using a xenograft mouse model injected with the highly glycolytic HCT116 colocarcinoma cells.

**Results:**

We demonstrate that the *bioenergetic signature *of isogenic HCT116 cancer cells inversely correlates with the potential to execute necrosis in response to 3BrP or IA treatment. Conversely, the *bioenergetic signature *directly correlates with the potential to execute apoptosis in response to 5-FU treatment in the same cells. However, despite the large differences observed in the *in vitro *cell-death responses associated with 3BrP, IA and 5-FU, the *in vivo *tumor regression activities of these agents were comparable.

**Conclusions:**

Overall, we suggest that the determination of the *bioenergetic signature *of colon carcinomas could provide a tool for predicting the therapeutic response to various chemotherapeutic strategies aimed at combating tumor progression.

## Background

Colorectal cancer (CRC) is a common neoplasia which poses a heavy burden on public health systems worldwide [1]. Despite the establishment of CRC screening protocols, tailored therapeutic approaches are required to minimize the significant social impact of this disease [1]. At present, *KRAS *mutation status is the only validated predictive marker for targeted CRC therapy [2]. Thus, the development and clinical implementation of new predictive molecular markers are needed to aid in the selection of patients likely to respond to therapy and rationalized CRC treatments [2].

Cancer cells and tumors have a predominant glycolytic metabolism, even under aerobic conditions [3,4]. Although the altered energetic metabolism of cancer cells has been proposed as a potential target for cancer treatment [3,5-7], it could also represent a therapeutic obstacle, because of its contribution to chemo- and radio-resistance [8]. In some tumors, this glycolytic phenotype is accompanied by a loss of bioenergetic activity in mitochondria [9,10], which can be estimated by determining its *bioenergetic signature *[10,11]. The *bioenergetic signature *is a protein ratio (β-F1-ATPase/GAPDH ratio) that assesses the expression of the catalytic subunit of mitochondrial H^+^-ATP synthase (β-F1-ATPase), a bottle-neck component required for the synthesis of biological energy, relative to the expression of glycolytic glyceraldehyde-3-phosphate dehydrogenase (GAPDH) [10]. Consistently, the *bioenergetic signature *has been observed to be significantly down-regulated in different human tumors compared to paired normal tissues [10,12-19]. Recent findings indicate that the *bioenergetic signature *also represents a functional index of metabolic activity because it correlates, both *in vivo *and *in vitro*, with the rate of glucose utilization by cancer cells and tumors [9,11]. Moreover, according to large cohort studies of colon [10,19], lung [9,14] and breast [16,20] cancer patients, low tumor *bioenergetic signatures *are associated with poor patient prognosis, strongly suggesting that impaired mitochondrial bioenergetics is at the heart of cancer progression.

Remarkably, down-regulation of β-F1-ATPase has been widely associated with the resistance of cancer cells to standard anticancer therapies [21-23]. In the specific case of colon cancer cells, chemotherapeutic response to 5-fluorouracil (5-FU) [11,21], as well as several metabolic inhibitors [23,24], was assessed in cells with different genetic backgrounds: a condition that is likely to affect the cellular response to chemotherapeutic agents. The recent development of isogenic HCT116 colon cancer cell lines, representing different *bioenergetic signatures *[11], has provided an opportunity to unambiguously assess the influence of energetic metabolism on colon cancer therapy. In this study, we investigated cell death responses in metabolically different isogenic HCT116 cells and the regression of tumor xenographs, in response to the glycolytic inhibitors 3-bromopyruvate (3BrP) and iodoacetate (IA), and the classic chemotherapeutic agent, 5-FU. The small alkylating 3BrP and IA target the enzymes of glycolysis hexokinase [25] and GAPDH [26], respectively, although recent findings suggest that 3BrP also targets GAPDH [27].

## Methods

### Cell cultures and treatments

Human colorectal carcinoma HCT116 cells were grown in McCoy's 5A media supplemented with 10% fetal bovine serum. Twenty four h after seeding, cells were left untreated (M-type), treated with 6 μM oligomycin (G-type), or treated with 10 mM 2-DG (SM-type) for 48h. On the day of the experiment, culture medium was replaced without the addition of any drug and cells were used at ~ 60% confluence for experiments. Where indicated, cells were incubated with 10 μM 5-FU for 48h, or 8 μM 3BrP or 100 μM IA for 7h.

### Protein electrophoresis and Western blot analysis

Cells were resuspended in lysis buffer (25 mM Hepes, 2.5 mM EDTA, 1% Triton X-100, 1 mM PMSF and 5 μg/mL leupeptin). Cell lysates were clarified by centrifugation at 11000 × g for 15 min. Resulting supernatants were fractionated on SDS-PAGE and transferred onto PVDF membranes for immunoblot analysis (Inmobilon-P, Millipore). Protein concentrations were determined using Bradford reagent (Bio-Rad protein assay). The primary monoclonal antibodies used were: anti-Hsp60 (Stressgene SPA-807, 1:2000) and anti-GAPDH (Abcam, 1:20000). The polyclonal rabbit anti-β-F1-ATPase (1:15000) [10] was also used. Peroxidase-conjugated anti-mouse or anti-rabbit IgGs (Nordic Immunonology, 1: 3000) were used as secondary antibodies. The blots were developed using the ECL reagent.

### Aerobic glycolysis

For determination of the rates of aerobic glycolysis, 0.1 mL aliquots of culture media were collected and used for enzymatic determination of lactate [11].

### Cell death assays

Exposure of phosphatidylserine on the cell surface was analyzed after various cellular treatments using the annexin V-FITC assay (Sigma-Aldrich). Briefly, cells were washed twice in PBS and incubated in the dark for 10 min at room temperature with FITC-conjugated annexin-V (50 μg/mL) and propidium iodide (100 μg/mL) solutions. For each analysis, 10,000 events were recorded in a FACScan (Becton-Dickinson). Cell death was also determined using fluorescence microscopy. In brief, cells treated with the different compounds described were harvested, washed with PBS and incubated in the dark for 5 min at room temperature with Hoechst 33342 (1 mg/mL) and propidium iodide (1 mg/mL) solutions. After washing, samples were observed under a Leica DM-IRB fluorescence microscope (UV). The percentage of dead (red stained) cells was calculated from 10-20 different randomly selected fields for each condition assayed.

### Caspase activity assays

Caspase 3/7 activity was determined using the luminogenic Ac-DEVD-pNA substrate included in the caspase-Glo 3/7 assay kit, according to the manufacturer's instructions (Promega). The reaction product was detected at 405 nm using a FLUOstar OPTIMA (BMG Labtech) plate luminometer.

### Determination of ATP

Approximately 6 × 10^4 ^cells were seeded and treated as indicated. Cellular ATP concentrations were determined using an ATP Bioluminiscence Assay Kit (Roche).

### In vivo tumorigenesis and treatments

Approximately, 1 × 10^7 ^G-type HCT116 cells were injected into the flank of 6-week-old male nude mice (National Cancer Institute, Frederick, Maryland). Tumor size was determined using a standard caliper and tumor volume was calculated using the formula: (width^2 ^× length) × 0.52, where width represents the shortest dimension of the tumor [11]. Twenty days after tumor induction, when tumors reached ~ 1,000 mm^3 ^of volume, animals were randomly allocated into four different groups for daily intraperitoneal injections (100 μL) with inhibitors of glycolysis (8 μM 3BrP or 100 μM IA), a conventional treatment for colon cancer (0.5 mM 5-FU) or 0.9% NaCl as a control group. All treatments were performed for six consecutive days. Following treatment, animals were weighted and killed and the tumors extracted. All animal experiments were conducted according to the ethical rules established by the Universidad Autónoma de Madrid Review Board.

### Statistical analysis

Statistical analysis was performed by Student's t test. Statistical tests were two-sided at the 5% level of significance. Pearson's correlation coefficient, p-value (*p*) and ANOVA with post hoc test (Dunnett's test) were calculated using the SPSS 17.0 software package.

## Results

**Figure 1:**
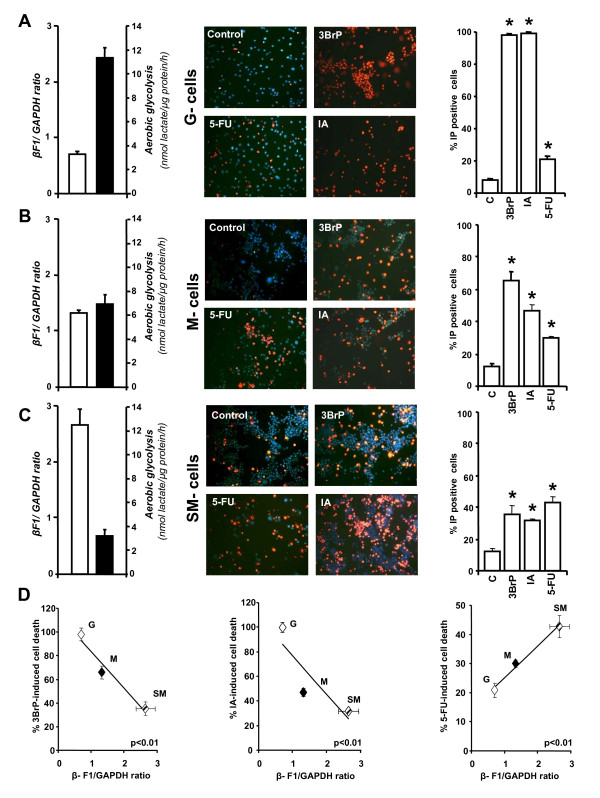
**The Bioenergetic Signature correlates with the cell-death response to chemotherapy **. HCT116 cells were treated as indicated [11] to produce cells with low (G-cells) **(A)**, medium (M-cells) **(B) **and high (SM-cells) **(C) ***bioenergetic signatures *(β-F1/GAPDH ratio). The rates of aerobic glycolysis in G-, M- and SM-cells are also indicated. Cells were exposed to the following agents: 8 μM 3BrP, 100 μM IA, 10 μM 5-FU or were left untreated (Control). Cells were double-stained with Hoechst 33342 and propidium iodide and visualized using fluorescence microscopy at 20x magnification. The percentage of dead cells (red cells, PI positive) was determined by examination of different randomly selected fields. Histograms shown **(A-C) **represent the means ± SEM of 10-25, 10-24 and 10-23 independent determinations in G-, M-, and SM-cells respectively. *, P < 0.05 for multiple comparisons by ANOVA and *post hoc *Dunnett's test. Plots in **(D) **illustrate the inverse (3BrP and IA) and direct (5-FU) correlation that exists between the *bioenergetic signature *of the cells and the death-response to the chemotherapeutic agents.

Because of the regulated expression of β-F1-ATPase, development of HCT116 colon cancer cell lines, displaying low (G-cells), medium (M-cells) or high (SM-cells) *bioenergetic signatures *(see Figure 1, and additional file 1) was accomplished by modification of cell culture conditions [11]. As recently detailed [11], the *bioenergetic signature *of each cell line was found to inversely correlate with the rate of aerobic glycolysis, where G-cells > M-cells > SM-cells (Figure 1A-C). Evaluation of cell death responses were assessed using fluorescence microscopy after double labeling with Hoechst 33342 and propidium iodide (PI) (Figure 1A-C). Our results show that death responses (% PI positive cells) to both metabolic inhibitors (3BrP and IA) decreased as the *bioenergetic signature *of the cells increased. Thus, the lower the *bioenergetic signature *of a cell the greater the death response to the glycolytic inhibitor treatment (G > M > SM) (Figure 1D). In fact, significant inverse correlations were uncovered between the *bioenergetic signature *of a cell and the extent of cell death following 3BrP (R = -0.633; n = 36, P < 0.01) and IA (R = -0.616; n = 36, P < 0.01) treatment, supporting the relevance of these glycolytic inhibitors in cancer treatment [7,23]. Specifically, in M-cells, 3BrP treatment was more effective than IA treatment at triggering cell death (Figure 1). In contrast, cell death in response to 5-FU treatment was found to directly correlate with *bioenergetic signature *(R = 0.519; n = 27, P < 0.01) (Figure 1D): as the activity of aerobic glycolysis is diminished cell death in response to 5-FU treatment is augmented (SM-cells > M-cells > G-cells), suggesting the participation of mitochondrial oxidative phosphorylation in the mechanism of 5-FU mediated cell death.

**Figure 2:**
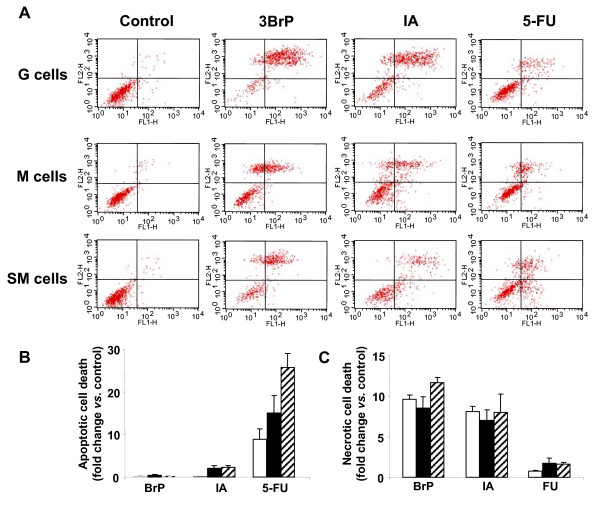
**The Bioenergetic Signature correlates with the cell-death pathway in response to chemotherapy **. HCT116 cells were treated as indicated [11] to produce cells with low (G-cells), medium (M-cells) and high (SM-cells) *bioenergetic signatures*. Cells were then treated with the following agents: 8 μM 3BrP, 100 μM IA, 10 μM 5-FU or left untreated (Control). A, Representative FACS analysis of cells after annexinV-FITC (50 μg/mL) and propidium iodide (100 μg/mL) staining are shown. The lower left quadrant corresponds to viable cells; the lower right quadrant early-apoptotic (annexin-positive) cells and the upper right and left quadrants corresponds to dead (PI positive) cells. Histograms shown are the means ± SEM of the percentage of apoptotic (annexin V positive) (B) and necrotic (PI positive) cells (C) from 4-6 independent determinations in G- (open bars), M- (closed bars) and SM-cells (hatched bars). *, p < 0.05 for multiple comparisons by ANOVA and *post hoc *Dunnett's test.

Flow cytometric analysis of plasma membrane exposure of phosphatidylserine (detected using an annexin V-FITC assay) was used as an index of apoptotic (annexin-positive) versus necrotic (PI positive) cell death [28] (Figure 2). This approach enables simultaneous estimation of the cell death pathway preferentially induced by each type of treatment. Upon treatment with the metabolic inhibitors 3BrP and IA, G-, M- and SM-cells all display a very large increase in the percentage of PI-positive cells (coupled with the absence of relevant changes in the percentage of annexin-positive cells) and thus appear to die by necrosis (Figure 2) [29,30]. In agreement with these results, no activation of caspase 3 (an apoptotic indicator) was observed following any of these above treatments in any of the cell lines tested (data not shown). Therefore, inhibition of the activity of glycolytic enzymes appears to trigger necrotic cell death. Furthermore, this effect was observed to be more pronounced in cells that rely more heavily on glycolysis as a pathway for energy provision. In contrast, 5-FU treatment of G-, M- and especially SM-cells resulted in a significant percentage of annexin-positive stained cells compared to controls (Figure 2), suggesting induction of apoptosis in response to 5-FU treatment. Importantly, this induction of apoptosis following 5-FU treatment appears to be more pronounced in cells that rely less heavily on glycolysis. In agreement with this finding, caspase 3 activity was found to be significantly increased in 5-FU treated SM-cells (1.0 ± 0.2 vs. 2.2 ± 0.1 a.u./15000 cells for control and 5-FU treated cells, respectively, P < 0.05). Therefore, efficient activation of apoptosis following 5-FU treatment may be associated with cellular reliance on mitochondrial energetic metabolism for cellular energy provision, in agreement with previous reports [11,21,31].

**Figure 3:**
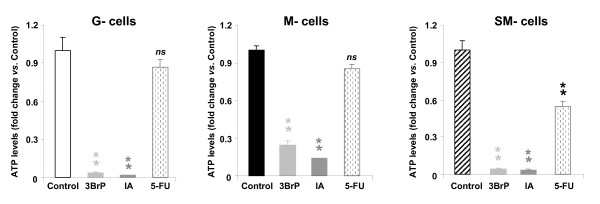
**Cellular ATP concentrations in response to chemotherapy **. HCT116 cells were treated as indicated [11] to produce cells with low (G-cells), medium (M-cells) and high (SM-cells) *bioenergetic signature*. Cells were then treated with the following agents: 8 μM 3BrP, 100 μM IA, 10 μM 5-FU or left untreated (Control) and ATP concentrations were determined. Histograms shown are means ± SEM. *, p < 0.05 compared to controls by Student's t test. *ns*, no significant.

To further confirm the cell death pathway activated in response to each of the treatments studied cellular ATP concentrations were determined (Figure 3). We observed that treatment of cells with 3BrP or IA was associated with a very large depletion of cellular ATP concentrations in all cell lineages (Figure 3), consistent with activation of necrosis by metabolic catastrophe in response to treatment with these metabolic inhibitors. In contrast, treatment of cells with 5-FU only marginally affected cellular ATP concentrations in G- and M-cells (Figure 3) and slightly, but significantly, promoted a 50% reduction in cellular ATP concentrations in SM-cells (Figure 3), indicating the absence of a compromised metabolic state following 5-FU treatment.

**Figure 4:**
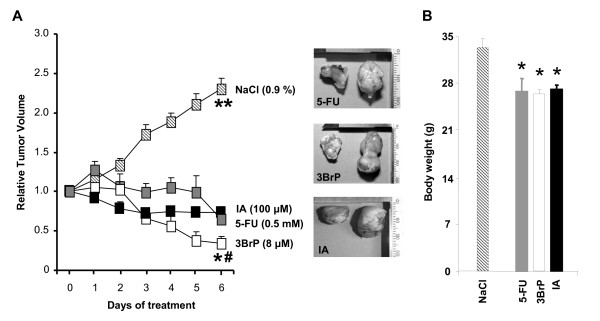
**Metabolic inhibitors effectively promote tumor regression **. HCT116 cells (10^7 ^cells per animal) with low *bioenergetic signature *(G-cells) were injected into nude mice for tumor development. Twenty days after, when tumor volume reached ~1 cm^3^, the animals received daily 100 μL intraperitoneal injections, containing 8 μM 3BrP (n = 10, open square), 0.5 mM 5-FU (n = 5, grey square) or 100 μM IA (n = 7, closed square) for six consecutive days. A 0.9% NaCl-treated control group (n = 6, hatched square) was also included for comparison. (A) Tumor volume is presented normalized to its volume before initiation of the treatments. * and #, p < 0.05 when comparing 3BrP with 5-FU- and IA-treated mice, respectively. **, p < 0.05 for multiple comparisons by ANOVA. Inserts provide representative examples of the differences in tumor size compared to controls. (B) Mice body weight (g) after treatments. Results shown are means ± SEM. *, p < 0.05 when compared to NaCl-treated controls by Student's t test.

Previous findings have suggested that only highly glycolytic G-cells are able to develop tumors in nude mice [11]. Therefore, in order to test the *in vivo *tumor regression activity of the metabolic inhibitors analyzed *in vitro*, animals were implanted with G-cells. Animals that developed ~ 1 cm^3 ^tumors were treated with daily doses of 8 μM 3BrP, 100 μM IA or 0.5 mM 5-FU over six consecutive days (Figure 4A). A control NaCl-treated group was also included for comparison (Figure 4A). Interestingly, from both a macroscopic (Figure 4B) and behavioral point of view, all treatments tested (except controls) seemed to affect the mice in a similar manner. Specifically, control animals developed a rapid 2.5-fold increase in tumor volume during the treatment period (Figure 4A). In contrast, animals treated with either 5-FU or IA revealed a significant ~ 30% decrease in tumor volume after 6 days of treatment (Figure 4A), while maximum tumor regression (> 50%) was observed in mice treated with 3BrP (Figure 4A), consistent with the higher cell-death trend associated with 3BrP treatment *in vitro *(Figure 1). However, the large differences in cell death triggered by 3BrP and IA compared to 5-FU in G-type cells (Figure 1A) were largely absent following *in vivo *treatments despite the fact that the tumors had a G-phenotype [11]. These results suggest that additional mechanisms may play a role in promoting tumor regression *in vivo *and that *in vitro *data should be extrapolated with caution.

## Discussion

In an effort to translate the *bioenergetic signature *to clinical practice, we have recently developed monoclonal antibodies against various markers of energetic metabolism [32]. We found that cancer abolishes cell-type specific differences in the *bioenergetic signature *[32], supporting its use as a generic target to combat different neoplasias [32]. Indeed, β-F1-ATPase expression has been shown to be a therapeutic response marker in different cancer cell lines, both for single and combined chemotherapy [19,21-24,33]. In the present study, we document the correlation between the *bioenergetic signature *of a cell, which represents an index of the relative relevance of cellular energy provision pathways [9,11], and the potential to execute cell death in response to the metabolic inhibitors, 3BrP and IA, and the anti-metabolite 5-FU. The correlations observed in this study cannot be ascribed to differences in the genetic background of the cells because: (i) all of the cells were derived from the same parental HCT116 cells and (ii) the energetic metabolism of HCT116 cells is a reversible phenotypic trait amenable to regulation [11,34]. Furthermore, although some cancer cells can oxidize glutamine for energy production purposes [3-5], glutamine contributes very little to the energetic metabolism of the highly glycolytic HCT116 cells used in this study. In fact, oxygen consumption rates, aerobic glycolysis rates and the bioenergetic signature of HCT116 cells are not affected by the presence of glutamine in the culture medium (see additional file 2).

Mechanistically, we propose that the cell death and tumor regression observed following administration of glycolytic inhibitors (3BrP and IA) may be due to induction of necrosis, whereas the cell death activity observed upon 5-FU treatment may occur through apoptosis (Figures 2 and 3). This later finding is consistent with the relevant roles played by oxidative phosphorylation [35] and mitochondrial H^+^-ATP synthase activity [33,36] in the efficient execution of cell death. Indeed, the bioenergetic activity of mitochondria in colon cancer cells [11,21] and tumors [19], has been associated with the ability to execute a ROS-mediated cell death response upon 5-FU treatment [11].

On the other hand, small alkylating agents have been shown to be able to kill cancer cells resistant to apoptosis by a process known as "programmed necrosis" through depletion of NAD^+ ^via PARP1 activation [30]. However, the induction of necrosis in response to the glycolytic inhibitors 3BrP and IA is exerted independently of PARP1 processing (data not shown), and most likely results from a metabolic catastrophe due to cellular ATP depletion (Figure 3) [23,24]. Overall, our studies suggest that the enzymes of glycolysis could represent therapeutic targets for the treatment of colon cancer that may be as effective as conventional treatments (5-FU) at promoting tumor regression, in agreement with findings by others [25,37].

The use of glycolytic inhibitors as chemotherapeutic agents has *pros *and *contras*. One problem is the deleterious effects that these agents could trigger in cell types strictly dependent on aerobic glycolysis for energy (e.g. neurons, lymphocytes, erythrocytes, retina, renal medulla, etc). However, glycolytic enzymes do have highly specific active site residues that, in principle, could provide more specific drug targets than those of proteins involved in signal transduction pathways. Thus, the use of such inhibitors may be beneficial in combination therapy as enhancers of the action of current chemotherapeutic drugs [7,23]. Targeting energetic metabolism might represent an alternative cancer treatment route in the near future, because tumor cells that are resistant to chemotherapy could effectively die by necrosis in response to different metabolic inhibitors. Whatever the case, the *bioenergetic signature *offers a reliable gauge to predict the cell death response (apoptotic or necrotic cell death) to cancer therapy.

## Conclusions

In summary, we have demonstrated that the *bioenergetic signature *of colon cancer cells inversely correlates with the potential to execute necrosis in response to treatments with glycolytic inhibitors. In contrast, the *bioenergetic signature *directly correlates with the apoptotic response to 5-FU treatment. Overall, our results support the use of the *bioenergetic signature *as a gauge for predicting cell death in response to different therapeutic strategies in colon cancer.

## List of abbreviations

β-F1-ATPase: β catalytic subunit of the mitochondrial H^+^-ATP synthase; *bioenergetic signature*: β-F1-ATPase/GAPDH ratio; GAPDH: Glyceraldehyde 3-phosphate dehydrogenase; IA: iodoacetate; 3BrP: 3-bromopyruvate; 5-FU: 5-fluorouracil; PI: propidium iodide; CRC: colorectal cancer.

## Competing interests

JMC as inventor and the Universidad Autónoma de Madrid hold the following patents on "the bioenergetic signature of cancer", which has been licensed to Fina Biotech, S.L. (Spain): US 10/514.771, Japanese 4235610, Canadian 2,487,176 and EU 03 727 509.6. MSA declares no competing interests.

## Authors' contributions

MSA carried out experiments. MSA and JMC designed experiments and wrote the manuscript. All authors read and approved the final manuscript.

## Supplementary Material

Additional file 1 **The bioenergetic signature of HCT116-derived cell lines. Representative western blot analysis**. Representative western blots of the expression of β-F1-ATPase, Hsp60 and GAPDH in two different preparations (lanes 1-2) of **(A) **2DG-treated (SM) and **(B) **OL-treated (G) cells when compared to non-treated (M) HCT116 cells. Click here for file

Additional file 2 **Effect of glutamine (Gln) in the energetic metabolism of HCT116 cells**. **(A) **Representative western blots of the expression of β- F1-ATPase, Hsp60 and GAPDH in two different preparations of HCT116 cells grown in the presence (+) or absence (-) of glutamine (Gln). The histogram illustrates the *bioenergetic signature *(β-F1/GAPDH ratio) in the presence (open bar) or absence (closed bar) of glutamine. **(B) **HCT116 cells were processed for the determination of the rates of aerobic glycolysis in the presence (open bar) or absence (closed bar) of glutamine. The rates of aerobic glycolysis were also determined after the addition of 6 μM oligomycin (hatched bars). **(C) **Determination of the rates of oxygen consumption. The results shown are the mean ± SEM of 6-15 independent determinations. No statistical significant differences were observed by Student's t-test in any of the parameters determined. Click here for file

## References

[B1] Gellad ZF Provenzale D Colorectal cancer: national and international perspective on the burden of disease and public health impact Gastroenterology 2010 138 2177 2190 10.1053/j.gastro.2010.01.056 20420954

[B2] Cunningham D Atkin W Lenz HJ Lynch HT Minsky B Nordlinger B Starling N Colorectal cancer Lancet 2010 375 1030 1047 10.1016/S0140-6736(10)60353-4 20304247

[B3] Cuezva JM Ortega AD Willers I Sanchez-Cenizo L Aldea M Sanchez-Arago M The tumor suppressor function of mitochondria: Translation into the clinics Biochim Biophys Acta 2009 1792 1145 1158 19419707 10.1016/j.bbadis.2009.01.006

[B4] DeBerardinis RJ Lum JJ Hatzivassiliou G Thompson CB The biology of cancer: metabolic reprogramming fuels cell growth and proliferation Cell Metab 2008 7 11 20 10.1016/j.cmet.2007.10.002 18177721

[B5] Kroemer G Pouyssegur J Tumor cell metabolism: cancer's Achilles' heel Cancer Cell 2008 13 472 482 10.1016/j.ccr.2008.05.005 18538731

[B6] Tennant DA Duran RV Gottlieb E Targeting metabolic transformation for cancer therapy Nat Rev Cancer 2010 10 267 277 10.1038/nrc2817 20300106

[B7] Pelicano H Martin DS Xu RH Huang P Glycolysis inhibition for anticancer treatment Oncogene 2006 25 4633 4646 10.1038/sj.onc.1209597 16892078

[B8] Diehn M Cho RW Lobo NA Kalisky T Dorie MJ Kulp AN Qian D Lam JS Ailles LE Wong M Association of reactive oxygen species levels and radioresistance in cancer stem cells Nature 2009 458 780 783 10.1038/nature07733 19194462 PMC2778612

[B9] Lopez-Rios F Sanchez-Arago M Garcia-Garcia E Ortega AD Berrendero JR Pozo-Rodriguez F Lopez-Encuentra A Ballestin C Cuezva JM Loss of the mitochondrial bioenergetic capacity underlies the glucose avidity of carcinomas Cancer Res 2007 67 9013 9017 10.1158/0008-5472.CAN-07-1678 17909002

[B10] Cuezva JM Krajewska M de Heredia ML Krajewski S Santamaria G Kim H Zapata JM Marusawa H Chamorro M Reed JC The bioenergetic signature of cancer: a marker of tumor progression Cancer Res 2002 62 6674 6681 12438266

[B11] Sanchez-Arago M Chamorro M Cuezva JM Selection of cancer cells with repressed mitochondria triggers colon cancer progression Carcinogenesis 2010 31 567 576 10.1093/carcin/bgq012 20080835

[B12] Bi X Lin Q Foo TW Joshi S You T Shen HM Ong CN Cheah PY Eu KW Hew CL Proteomic analysis of colorectal cancer reveals alterations in metabolic pathways: mechanism of tumorigenesis Mol Cell Proteomics 2006 5 1119 1130 10.1074/mcp.M500432-MCP200 16554294

[B13] Chen J Kahne T Rocken C Gotze T Yu J Sung JJ Chen M Hu P Malfertheiner P Ebert MP Proteome analysis of gastric cancer metastasis by two-dimensional gel electrophoresis and matrix assisted laser desorption/ionization-mass spectrometry for identification of metastasis-related proteins J Proteome Res 2004 3 1009 1016 10.1021/pr049916l 15473690

[B14] Cuezva JM Chen G Alonso AM Isidoro A Misek DE Hanash SM Beer DG The bioenergetic signature of lung adenocarcinomas is a molecular marker of cancer diagnosis and prognosis Carcinogenesis 2004 25 1157 1163 10.1093/carcin/bgh113 14963017

[B15] He QY Chen J Kung HF Yuen AP Chiu JF Identification of tumor-associated proteins in oral tongue squamous cell carcinoma by proteomics Proteomics 2004 4 271 278 10.1002/pmic.200300550 14730689

[B16] Isidoro A Casado E Redondo A Acebo P Espinosa E Alonso AM Cejas P Hardisson D Fresno Vara JA Belda-Iniesta C Breast carcinomas fulfill the Warburg hypothesis and provide metabolic markers of cancer prognosis Carcinogenesis 2005 26 2095 2104 10.1093/carcin/bgi188 16033770

[B17] Isidoro A Martinez M Fernandez PL Ortega AD Santamaria G Chamorro M Reed JC Cuezva JM Alteration of the bioenergetic phenotype of mitochondria is a hallmark of breast, gastric, lung and oesophageal cancer Biochem J 2004 378 17 20 10.1042/BJ20031541 14683524 PMC1223948

[B18] Unwin RD Craven RA Harnden P Hanrahan S Totty N Knowles M Eardley I Selby PJ Banks RE Proteomic changes in renal cancer and co-ordinate demonstration of both the glycolytic and mitochondrial aspects of the Warburg effect Proteomics 2003 3 1620 1632 10.1002/pmic.200300464 12923786

[B19] Lin PC Lin JK Yang SH Wang HS Li AF Chang SC Expression of beta-F1-ATPase and mitochondrial transcription factor A and the change in mitochondrial DNA content in colorectal cancer: clinical data analysis and evidence from an in vitro study Int J Colorectal Dis 2008 23 1223 1232 10.1007/s00384-008-0539-4 18769884

[B20] Ortega AD Sala S Espinosa E Gonzalez-Baron M Cuezva JM HuR and the bioenergetic signature of breast cancer: a low tumor expression of the RNA-binding protein predicts a higher risk of disease recurrence Carcinogenesis 2008 29 2053 2061 10.1093/carcin/bgn185 18687667

[B21] Shin YK Yoo BC Chang HJ Jeon E Hong SH Jung MS Lim SJ Park JG Down-regulation of mitochondrial F1F0-ATP synthase in human colon cancer cells with induced 5-fluorouracil resistance Cancer Res 2005 65 3162 3170 15833846 10.1158/0008-5472.CAN-04-3300

[B22] Li RJ Zhang GS Chen YH Zhu JF Lu QJ Gong FJ Kuang WY Down-regulation of mitochondrial ATPase by hypermethylation mechanism in chronic myeloid leukemia is associated with multidrug resistance Ann Oncol 2010 7 1506 1514 10.1093/annonc/mdp569 20038517

[B23] Hernlund E Hjerpe E Avall-Lundqvist E Shoshan M Ovarian carcinoma cells with low levels of beta-F1-ATPase are sensitive to combined platinum and 2-deoxy-D-glucose treatment Mol Cancer Ther 2009 8 1916 1923 10.1158/1535-7163.MCT-09-0179 19567816

[B24] Hernlund E Ihrlund LS Khan O Ates YO Linder S Panaretakis T Shoshan MC Potentiation of chemotherapeutic drugs by energy metabolism inhibitors 2-deoxyglucose and etomoxir Int J Cancer 2008 123 476 483 10.1002/ijc.23525 18452174

[B25] Geschwind JF Ko YH Torbenson MS Magee C Pedersen PL Novel therapy for liver cancer: direct intraarterial injection of a potent inhibitor of ATP production Cancer Res 2002 62 3909 3913 12124317

[B26] Bickis IJ Quastel JH Effects of Metabolic Inhibitors on Energy Metabolism of Ehrlich Ascites Carcinoma Cells Nature 1965 205 44 46 10.1038/205044a0 14283130

[B27] Ganapathy-Kanniappan S Geschwind JF Kunjithapatham R Buijs M Vossen JA Tchernyshyov I Cole RN Syed LH Rao PP Ota S Vali M Glyceraldehyde-3-phosphate dehydrogenase (GAPDH) is pyruvylated during 3-bromopyruvate mediated cancer cell death Anticancer Res 2009 29 4909 4918 20044597 PMC3743725

[B28] Pigault C Follenius-Wund A Schmutz M Freyssinet JM Brisson A Formation of two-dimensional arrays of annexin V on phosphatidylserine-containing liposomes J Mol Biol 1994 236 199 208 10.1006/jmbi.1994.1129 8107105

[B29] Jaattela M Multiple cell death pathways as regulators of tumour initiation and progression Oncogene 2004 23 2746 2756 10.1038/sj.onc.1207513 15077138

[B30] Zong WX Ditsworth D Bauer DE Wang ZQ Thompson CB Alkylating DNA damage stimulates a regulated form of necrotic cell death Genes Dev 2004 18 1272 1282 10.1101/gad.1199904 15145826 PMC420353

[B31] Munoz-Pinedo C Ruiz-Ruiz C Ruiz de Almodovar C Palacios C Lopez-Rivas A Inhibition of glucose metabolism sensitizes tumor cells to death receptor-triggered apoptosis through enhancement of death-inducing signaling complex formation and apical procaspase-8 processing J Biol Chem 2003 278 12759 12768 10.1074/jbc.M212392200 12556444

[B32] Acebo P Giner D Calvo P Blanco-Rivero A Ortega AD Fernandez PL Roncador G Fernandez-Malave E Chamorro M Cuezva JM Cancer abolishes the tissue type-specific differences in the phenotype of energetic metabolism Transl Oncol 2009 2 138 145 19701498 10.1593/tlo.09106 PMC2730139

[B33] Santamaria G Martinez-Diez M Fabregat I Cuezva JM Efficient execution of cell death in non-glycolytic cells requires the generation of ROS controlled by the activity of mitochondrial H+-ATP synthase Carcinogenesis 2006 27 925 935 10.1093/carcin/bgi315 16361271

[B34] Rossignol R Gilkerson R Aggeler R Yamagata K Remington SJ Capaldi RA Energy substrate modulates mitochondrial structure and oxidative capacity in cancer cells Cancer Res 2004 64 985 993 10.1158/0008-5472.CAN-03-1101 14871829

[B35] Tomiyama A Serizawa S Tachibana K Sakurada K Samejima H Kuchino Y Kitanaka C Critical role for mitochondrial oxidative phosphorylation in the activation of tumor suppressors Bax and Bak J Natl Cancer Inst 2006 98 1462 1473 10.1093/jnci/djj395 17047195

[B36] Matsuyama S Xu Q Velours J Reed JC The Mitochondrial F0F1-ATPase proton pump is required for function of the proapoptotic protein Bax in yeast and mammalian cells Mol Cell 1998 1 327 336 10.1016/S1097-2765(00)80033-7 9660917

[B37] Fantin VR St-Pierre J Leder P Attenuation of LDH-A expression uncovers a link between glycolysis, mitochondrial physiology, and tumor maintenance Cancer Cell 2006 9 425 434 10.1016/j.ccr.2006.04.023 16766262

