# Supplementary material for: The bioenergetic signature of isogenic colon cancer cells predicts the cell death response to treatment with 3-bromopyruvate, iodoacetate or 5-fluorouracil
Source: J Transl Med. 2011 Feb 8;9:19. doi: 10.1186/1479-5876-9-19 (PMC3045315; doi:10.1186/1479-5876-9-19)
Supplement: Additional file 1 — The bioenergetic signature of HCT116-derived cell lines. Representative western blot analysis. Representative western blots of the expression of β-F1-ATPase, Hsp60 and GAPDH in two different preparations (lanes 1-2) of (A) 2DG-treated (SM) and (B) OL-treated (G) cells when compared to non-treated (M) HCT116 cells. [file 1479-5876-9-19-S1.PDF]

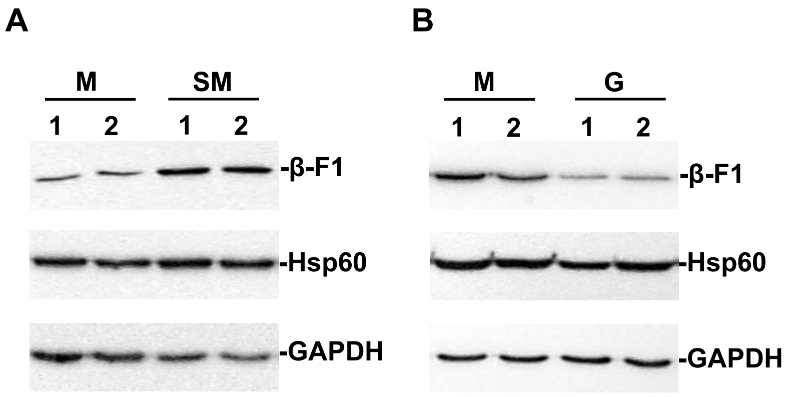

Additional file 1. The *bioenergetic signature* of HCT116-derived cells. Representative western blots of the expression of β-F1-ATPase, Hsp60 and GAPDH in two different preparations (lanes 1-2) of **(A)** 2DG-treated (SM) and **(B)** OL-treated (G) cells when compared to non-treated (M) HCT116 cells.
