# Supplementary material for: The bioenergetic signature of isogenic colon cancer cells predicts the cell death response to treatment with 3-bromopyruvate, iodoacetate or 5-fluorouracil
Source: J Transl Med. 2011 Feb 8;9:19. doi: 10.1186/1479-5876-9-19 (PMC3045315; doi:10.1186/1479-5876-9-19)
Supplement: Additional file 2 — Effect of glutamine (Gln) in the energetic metabolism of HCT116 cells. (A) Representative western blots of the expression of β- F1-ATPase, Hsp60 and GAPDH in two different preparations of HCT116 cells grown in the presence (+) or absence (-) of glutamine (Gln). The histogram illustrates the bioenergetic signature (β-F1/GAPDH ratio) in the presence (open bar) or absence (closed bar) of glutamine. (B) HCT116 cells were processed for the determination of the rates of aerobic glycolysis in the presence (open bar) or absence (closed bar) of glutamine. The rates of aerobic glycolysis were also determined after the addition of 6 μM oligomycin (hatched bars). (C) Determination of the rates of oxygen consumption. The results shown are the mean ± SEM of 6-15 independent determinations. No statistical significant differences were observed by Student's t-test in any of the parameters determined. [file 1479-5876-9-19-S2.PDF]

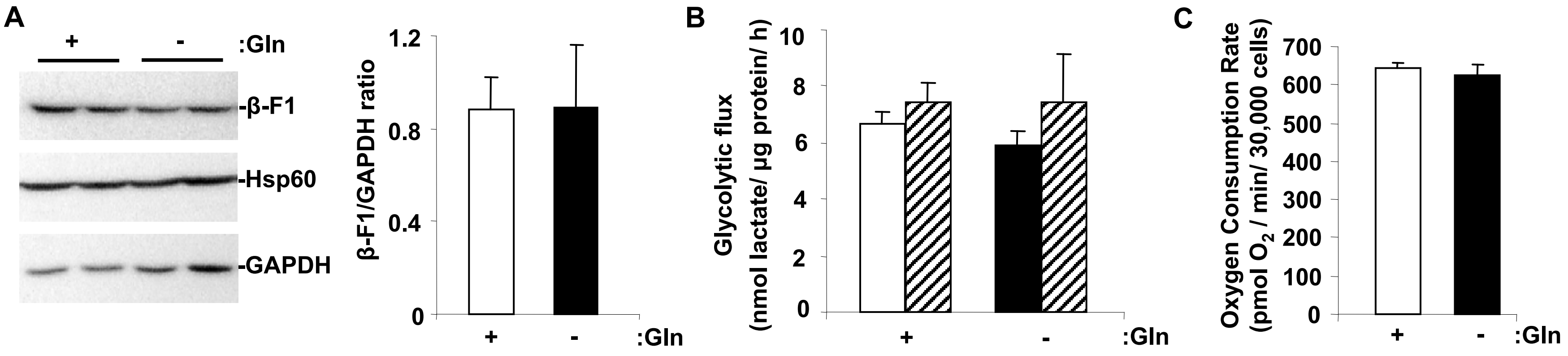

Additional file 2. Effect of glutamine (Gln) in the energetic metabolism of HCT116 cells. (A) Representative western blots of the expression of β-F1-ATPase, Hsp60 and GAPDH in two different preparations of HCT116 cells grown in the presence (+) or absence (-) of glutamine (Gln). The histogram illustrates the *bioenergetic signature* (β-F1/GAPDH ratio) in the presence (open bar) or absence (closed bar) of glutamine. **(B)** HCT116 cells were processed for the determination of the rates of aerobic glycolysis in the presence (open bar) or absence (closed bar) of glutamine. The rates of aerobic glycolysis were also determined after the addition of 6µM oligomycin (hatched bars). **(C)** Determination of the rates of oxygen consumption. The results shown are the mean ± SEM of 6-15 independent determinations. No statistical significant differences were observed by Student's t-test in any of the parameters determined.
